# An insight into the role of the organic acids produced by *Enterobacter* sp. strain 15S in solubilizing tricalcium phosphate: in situ study on cucumber

**DOI:** 10.1186/s12866-023-02918-6

**Published:** 2023-07-12

**Authors:** Mónica Yorlady Alzate Zuluaga, André Luiz Martinez de Oliveira, Fabio Valentinuzzi, Nádia Souza Jayme, Sonia Monterisi, Roberto Fattorini, Stefano Cesco, Youry Pii

**Affiliations:** 1 Faculty of Agricultural, Environmental and Food Sciences, Free University of Bolzano, Piazza Università 5, Bolzano, 39100 Italy; 2 Department of Biochemistry and Biotechnology, State University of Londrina, Londrina, Paraná, Brazil

**Keywords:** Phosphate solubilization, Phosphate-solubilizing bacteria (PSB), *Enterobacter* sp., Organic acids, P-use efficiency

## Abstract

**Background:**

The release of organic acids (OAs) is considered the main mechanism used by phosphate-solubilizing bacteria (PSB) to dissolve inorganic phosphate in soil. Nevertheless, little is known about the effect of individual OAs produced by a particular PSB in a soil–plant system. For these reasons, the present work aimed at investigating the effect of *Enterobacter* sp. strain 15S and the exogenous application of its OAs on (i) the solubilization of tricalcium phosphate (TCP), (ii) plant growth and (iii) P nutrition of cucumber. To this purpose two independent experiments have been performed.

**Results:**

In the first experiment, carried out in vitro, the phosphate solubilizing activity of *Enterobacter* 15S was associated with the release of citric, fumaric, ketoglutaric, malic, and oxalic acids. In the second experiment, cucumber plants were grown in a Leonard jar system consisting of a nutrient solution supplemented with the OAs previously identified in *Enterobacter* 15S (jar’s base) and a substrate supplemented with the insoluble TCP where cucumber plants were grown (jar’s top). The use of *Enterobacter* 15S and its secreted OAs proved to be efficient in the in situ TCP solubilization. In particular, the enhancement of the morpho-physiological traits of P-starved cucumber plants was evident when treated with *Enterobacter* 15S, oxalate, or citrate. The highest accumulation of P in roots and shoots induced by such treatments further corroborated this hypothesis.

**Conclusion:**

In our study, the results presented suggest that organic acids released by *Enterobacter* 15S as well as the bacterium itself can enhance the P-acquisition by cucumber plants.

**Supplementary Information:**

The online version contains supplementary material available at 10.1186/s12866-023-02918-6.

## Introduction

Phosphorus (P) is an essential macronutrient required for plant development and productivity [1]. However, compared to other essential nutrients, P is poorly mobile in soil and little available to plants in most pedological conditions [2]. Consequently, increasing amounts of fertilizers are annually added (16.5 million tons of P globally) to maintain the available P fraction at optimal levels for an appropriate agricultural productivity [3].

In soil, P is present in a wide range of organic and inorganic forms accounting for 30–50% and 35–70% of the total P, respectively [4]. In acidic soils, inorganic P is commonly found as insoluble mineral compounds, like iron (Fe) and aluminum (Al) phosphates, or it can be incorporated into rocks rich in mineral oxides. In alkaline soils, inorganic P is trapped as insoluble calcium (Ca) phosphate compounds [5]. Plants naturally take up P from soil solutions either as H_2_PO_4_^−^, available at pH < 7.2, or as HPO_4_^2−^, at pH > 7.2 [6]. However, in most of the cases, P is strongly adsorbed on soil particles and readily retained in soil. Therefore, only 0.1% of P in soil is generally present in an available form for plant acquisition [7]. In this regard, intensive use of P fertilizers, most of which undergo precipitation processes, may result in excessive P accumulation in agricultural soils with serious environmental and ecological threats.

Nowadays, the development of innovative strategies aimed at reducing the rate of P-based fertilizers application in crop systems is gaining considerable attention. These strategies include i) intercropping with appropriate plant species able to favor P mobilization in soil; ii) tissue-specific overexpression in plants of homologous genes involved in P uptake, translocation and utilization; and iii) the use of beneficial microorganisms for efficient P mining and solubilization favoring then, in turn, a more efficient plant acquisition of the nutrient [8]. With respect to the last one, phosphorus solubilizing bacteria (PSB), which are commonly found in bulk soil and in the rhizosphere of most plants, are at present attracting particular attention for their abilities to improve plant P acquisition as well as development and productivity [9]. There are several examples of this type in literature such as those using *Pseudomonas* [10, 11], *Bacillus* [12], *Enterobacter* [9, 13], *Burkholderia* [14], *Pantoea* [15], *Acinetobacter* [16].

In soil, the PSB-mediated solubilization of mineral P fraction is associated with the secretion of phenolic compounds, siderophores, organic acids and protons, with these latter causing a drop in the soil pH with a consequent P mobilization from the mineral sources [17]. On the other hand, organic acids (OAs), due to carboxyl and hydroxyl functional groups, show a high affinity for cations like Ca^+2^, Fe^+3^ and Al^+3^ thus favoring the P mobilization via a ligand exchange reactions [18]. However, the efficiency of the PSB-dependent P solubilization varies as a function of the amount, strength and type of OAs released [19]. For instance, tri- and dicarboxylic acids (*e.g*., citric, fumaric, glutaric, ketoglutaric, malic, malonic, succinic, oxalic, tartaric) are more efficient in P solubilization than monobasic acids (*e.g*., acetic, gluconic, glycolic, lactic) [20]. Moreover, Gram-negative bacteria, due to their greatest ability of producing OAs, appear to be more effective at dissolving mineral phosphates than Gram-positive ones [21].

It is well known that OA synthesis and release by PSB strains is independent of their genetic relatedness; furthermore, each strain has its own ability of secreting OAs during the solubilization of inorganic P [22]. Therefore, different mixtures of OAs have been identified according to the bacterial species grown in liquid media supplemented with Ca_3_(PO_4_)_2_. For instance, the most commonly OAs produced by the strain EnHy-401 of *Enterobacter* sp. were oxalic, gluconic, malic, lactic, citric, succinic and fumaric acids [23]. On the contrary *Burkholderia multivorans* produced oxalic, gluconic, shikimic and lactic acids [24], whilst *Bacillus* sp. strain MVY produced mixtures of citric, succinic, 2-ketogluconic, gluconic, malic, lactic, and oxalic acid [25].

For this reason, there is a need of understanding how and to which extent the different OAs secreted by a particular PSB may contribute to P solubilization in soils, and, consequently, to plant P nutrition. Several experiments have been focused on the qualitative identification of OAs produced by a large diversity of PSB (see the review by Timofeeva et al. [20]), whereas other studies have investigated either the effect of OAs on P mobilization in soil, yet under in vitro conditions [26, 27], or on the biochemical and biological properties of soil [28]. Other authors have reported the role of OAs as plant biostimulants when foliarly applied (see book chapter by Hadavi and Ghazijahani [29]), whereas few studies have evaluated the effect of external addition of OAs on plant growth and P uptake [30–32]. However, to the best of our knowledge, the effects of the individual OAs produced by a particular bacterial strain in a soil–plant system have never been described yet. This lack of information is crucial not only from the knowledge advancement but also for a wider-scale exploitation of the benefits that could derive from the use of these microorganisms in a context of more sustainable agricultural practice.

Based on these premises, the present work was aimed at investigating the mechanisms underpinning the P solubilization ability of *Enterobacter* sp. strain 15S. To achieve this objective, two independent experiments have been carried out. In the first one, performed in vitro, the TCP solubilization and OAs production by *Enterobacter* 15S have been evaluated to identify the main effectors of P solubilization for this specific strain. In the second one, performed under in situ conditions, the effects of both *Enterobacter* 15S inoculation and amendment with different OAs on TCP solubilization, cucumber plant development, and P accumulation under limited P availability have been investigated. Plant development, mineral elements accumulated in tissues, P use efficiency by plants, and the transcriptional modulation of the P transporter gene *CsPT1.4* have been monitored and discussed.

## Materials and methods

### Experiment 1: In vitro assay with the bacterial strain *Enterobacter* sp. 15S

#### Bacterial inoculant

*Enterobacter* sp. strain 15S (KX884932.1) was selected for its potential in solubilizing different sources of insoluble P [9, 13]. The bacterial strain was grown in Luria–Bertani (LB) medium under orbital shaking at 180 rpm, 28 °C for 24 h. Afterward, cells were washed and re-suspended in a sterile saline solution (0.85% w/v NaCl) to a standard concentration of 10^8^ cell mL^−1^ that was used as inoculant for the planned experiments.

#### Tricalcium phosphate solubilization assay

The inorganic phosphate solubilization ability of *Enterobacter* sp. 15S was evaluated using the liquid NBRIP medium [33] supplemented with Ca_3_(PO_4_)_2_ at 0.1% P (w/v) and initial pH adjusted to 6.7. The experiment was carried out in 1 L Erlenmeyer flasks containing 300 mL of NBRIP medium. The bacterium was inoculated at a final concentration of 10^6^ cell mL^−1^ in triplicate and the flasks were incubated at 28 °C with shaking at 180 rpm for 7 days. A triplicate of non-inoculated flasks was used as negative control. Aliquots of bacterial culture were collected at initial time (before inoculation) and every 24 h after inoculation. Culture samples were centrifuged at 10,000 rpm for 10 min at 4 °C. The supernatant was filtered through a 0.22 µm Millipore filter, and used to estimate the solubilized phosphate, pH values and levels of OAs release.

#### Quantification of phosphate solubilization capacity in liquid culture

The phosphate solubilization capacity of *Enterobacter* 15S was evaluated in the supernatant by using the Fiske and Subbarow method [34]. The absorbance was measured at 660 nm and the amount of solubilized phosphate was extrapolated from a calibration curve obtained using KH_2_PO_4_ as standard. A digital pH meter was used to determine the pH of the supernatant.

#### Determination of organic acids (OAs)

The evaluation of OAs produced by *Enterobacter sp.* strain 15S was carried out according to Valentinuzzi et al. [35]. Briefly, The OAs were separated by HPLC on a cation exclusion column (Aminex HPX-87H 300 mm × 7.8 mm, Bio- Rad Laboratories Inc.) with 10 mM H_2_SO_4_ as mobile phase at a constant flow rate of 0.6 mL min^−1^. The OAs were detected at a wavelength of 210 nm (PDA 2998, Waters Spa, Italy). Standard acids were prepared as individual stock solutions using Sigma free acids and then combined to give diluted reference standards.

### Experiment 2: In situ assay using cucumber plants in Leonard jars system

**Fig. 1:**
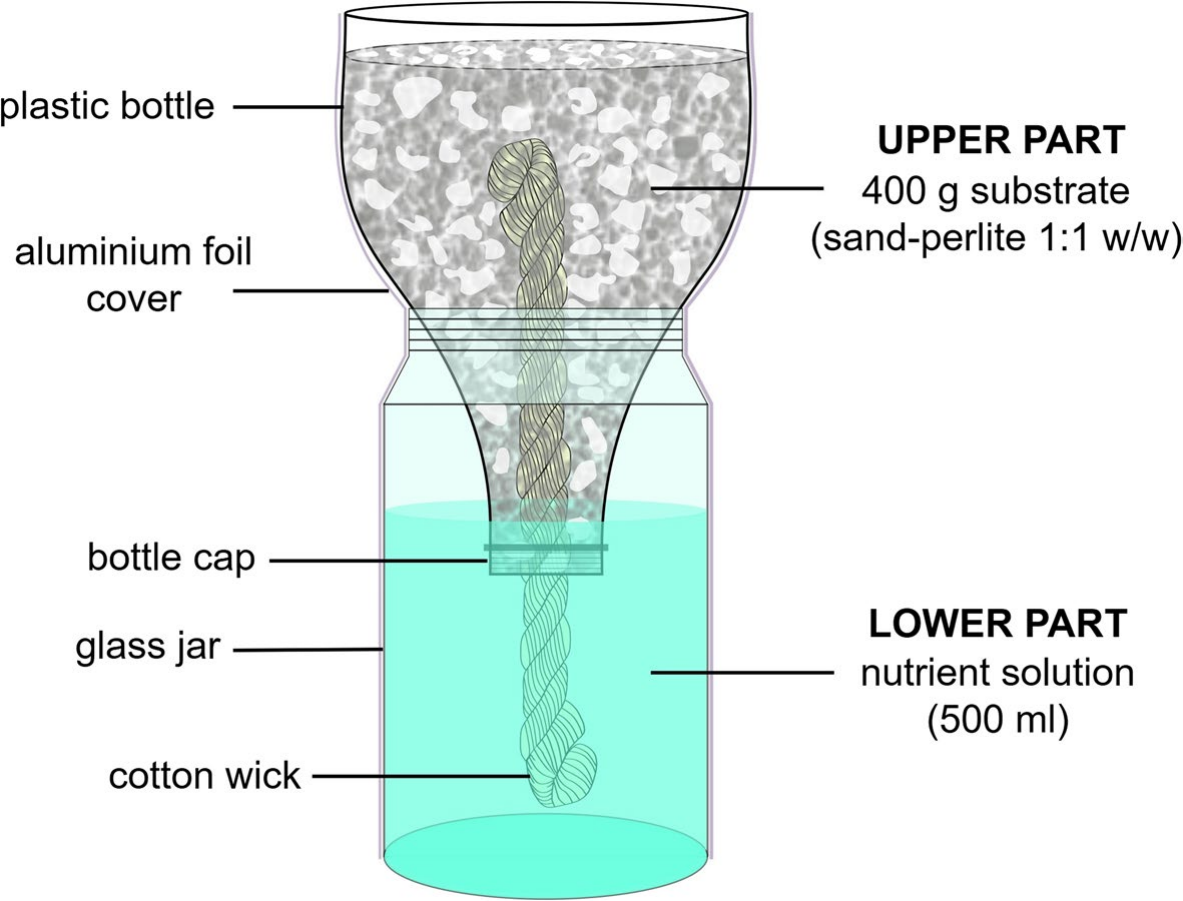
Schematic of the Leonard jar system used in this study

After performing the Experiment 1, the phosphate solubilizing ability of *Enterobacter* 15S was associated with the release of five OAs. In this sense, we decided to conduct an experiment to better understand the P solubilization mechanisms used by this bacterial strain in a soil–plant system. For this purpose, we compare the effect of the inoculation with *Enterobacter* with the use of either single OA alone or the mix of the five OAs at the same concentrations produced at the end of the Experiment 1. Thus, our intention was to simultaneously explore whether the concentrations of OAs produced under in vitro conditions could efficiently promote the release of P from soil when exogenously applied to a P-free substrate, yet supplemented with the inorganic phosphate Ca_3_(PO_4_)_2_, and/or help plant development and P nutrition. To avoid undesired P leaching losses, that are likely to happen during irrigation in a typical pot experiment [36], and to get a more representative estimation of the P released by the treatments, a modified Leonard jars system was adopted (Fig. 1). Briefly, our Leonard jars consisted of two separated parts: a lower part that provided a reservoir for nutrient solution (NS) either supplemented or not with the OAs (Table 1), and an upper part, supporting plant growth, that contained the substrate either supplemented or not with the insoluble phosphate Ca_3_(PO_4_)_2_ (Table 1). A cotton wick (20 cm long) connected both parts bringing up the NS from the base to the top by capillarity (Fig. 1). Additionally, two control treatments were adopted (Table 1): a positive control (Control P +), in which the amount of soluble P added (KH_2_PO_4_) via NS was equivalent to the P supplemented as insoluble Ca_3_(PO_4_)_2_; a negative control (Control P-), consisting of a P-free NS with neither OAs supplementation nor bacterial inoculation.

**Table 1:** Description of each treatment in this study

Treatment	Leonard jar system	
	**Lower part**	**Upper part**
15S	P-free NS	400 g of substrate + 2 g of Ca_3_(PO_4_)_2_ Inoculation by pipetting 1 ml of bacterial biostimulant *Enterobacter* sp. 15S (10^8^ cells ml^−1^) onto the seedlings at the rooting site
Ci	P-free NS supplemented with 1 mM citric acid	400 g of substrate + 2 g of Ca_3_(PO_4_)_2_
Fu	P-free NS supplemented with 1 mM fumaric acid	
Ke	P-free NS supplemented with 12 mM α-ketoglutaric acid	
Ma	P-free NS supplemented with 7 mM malic acid	
Ox	P-free NS supplemented with 2 mM oxalic acid	
Mix	P-free NS supplemented with 1 mM citric acid + 1 mM fumaric acid + 12 mM α-ketoglutaric acid + 7 mM malic acid + 2 mM oxalic acid	
Control P-	P-free NS	
Control P +	P-full NS (KH_2_PO_4_ 0.1% P w/v)	400 g of substrate

#### Plant growth conditions and experimental design

Cucumber (*Cucumis sativus* L. cv. Chinese Long) seeds were acquired from a local supplier (Biasion, Bolzano, Italy) and germinated for 5 days in the dark at 22 °C on filter paper moistened with 0.5 mM CaSO_4_. After the germination period, the cucumber seedlings were transferred to the upper part of Leonard jars system (one plant per jar) filled with 400 g of inert substrate obtained by mixing sand (Table S1) and perlite in a proportion of 1:1 (w/w) supplemented with 2 g of Ca_3_(PO_4_)_2_ (0.1% P w/w) (Fig. 1). The lower part of the Leonard jars was filled with 500 mL of P-free NS buffered at pH 6 with MES KOH 1 mM and containing 2 mM Ca (NO_3_)_2_, 0.7 mM K_2_SO_4_, 0.5 mM MgSO_4_, 0.1 mM KCl, 0.5 μM MnSO_4_, 10 μM H_3_BO_3_, 0.5 μM ZnSO_4_, 0.2 μM CuSO_4_, 0.01 μM (NH_4_)_6_Mo7O_24_, and 80 μM Fe-EDTA (Fig. 1). The experimental design comprised nine treatments with six biological replicates (six Leonard jars per treatment). Detailed information of each treatment is shown in Table 1. The NS in the lower part of Leonard jars was replaced every week. Plants were kept under controlled environmental conditions in a climatic chamber 14/10 h day/night, 24/19 °C, 250 µmol m^−2^ s^−1^ light intensity and 70% relative humidity. After 21 days of cultivation, plants were harvested and analyzed as described below. Three biological replicates of each treatment were used to assess morpho-physiological parameters and elemental analysis. The other three biological replicates were used for gene expression analysis.

The same experimental design (Table 1) was performed without cucumber plants in order to assess the available phosphorus in the substrate after 21 days of experiment. The available P (*i.e.,* amount of Ca_3_(PO_4_)_2_ solubilized) was determined according to Bolle et al. [37]. Briefly, two grams of substrate and 20 ml of distilled water were shaken for 1 h. Thereafter, samples were centrifuged at 10000 rpm for 10 min, followed by filtration through Whatman filter paper. The directly plant-available P was determined by the Fiske and Subbarow method [34].

#### Characterization of plant growth parameters

At harvest, the soil–plant analysis development (SPAD) units were measured by a chlorophyll meter (model SPAD-502; Minolta, Osaka, Japan). The leaf area was measured on all the leaves using a leaf area meter (model LI-3000C; LiCOR, Lincoln, NE, USA) and used to calculate the total leaf area of a plant by summing the areas of individual leaves. Roots and shoots were separated and dried at 65 °C until constant weight. The dry weight (DW) of the roots and shoots was recorded.

#### Elemental analysis

Dry root and leaf tissues of cucumber plants were digested with 69% ultrapure HNO_3_ in a single reaction chamber microwave digestion system (UltraWAVE, Milestone, Shelton, CT, USA). An inductively coupled plasma–mass spectrometer (ICP-MS, iCAP™ RQ, Thermo Scientific) was used for the multi-element analysis. Element quantification was carried out using certified multi-element standards (CPI International, https://cpiinternational.com). The accuracy of the analysis was assessed using certified reference materials 1573a (tomato leaves) and 1570a (spinach leaves).

#### Gene expression analysis

Root tissues from plants of each treatment were collected, frozen in liquid N and stored at -80 °C until use. Total RNA was extracted using the Spectrum Plant Total RNA Kit (Sigma-Aldrich, St. Louis, MO, USA) according to the manufacturer’s instructions. The reverse transcription was performed using the ImProm-II Reverse Transcription System (Promega, Madison, WI, USA) following the manufacturer’s instructions. Gene-specific primers were designed for the target gene *CsPT1.4* and the housekeeping gene *CsEF1α* (Table S2). Quantitative real-time reverse transcription PCR (qRT-PCR) was carried out in triplicate as following the conditions described previously [9]. The relative expression ratio values were calculated by using the 2^−ΔΔCt^ method according to Livak and Schmittgen [38].

#### Indexes calculation

The following P-efficiency indexes were calculated to establish the relationship between P in the different treatments and plant development of cucumber plants, *i.e*., P accumulated in roots (PAR) and shoots (PAS), utilization efficiency (PUE), and apparent recovery efficiency (AREP) adapted from the methodology described by Oliveira et al. [39]:

- Phosphorus accumulated in roots (PAR)
  $$PAR\;\left(mg\right)=P\;concentration\left(mg\;{g\;root}^{-1}\right)\times root\;dry\;weight\;{(g\;root}^{-1})$$
- Phosphorus accumulated in shoots (PAS)
  $$PAS\;\left(mg\right)=P\;concentration\;\left(mg\;{g\;shoot}^{-1}\right)\times shoot\;dry\;weight\;{(g\;shoot}^{-1})$$
- Phosphorus utilization efficiency (PUE)
  $$PUE\;\left(\%\right)=\left(\frac{{Shoot\;dry\;weight}_{treatment}-{Shoot\;dry\;weight}_{Control\;P-}}{{Available\;P}_{treatment}}\right)\times100$$
- Apparent recovery efficiency of phosphorus (AREP)
  $$AREP\;\left(\%\right)=\left(\frac{{PAS}_{treatment}-{PAS}_{Control\;P-}}{{Available\;P}_{treatment}}\right)\times100$$

### Statistical analysis

The results are presented as means of three replicates ± standard errors (SE). One-way analysis of the variance (ANOVA) was performed using R software (version 4.0.3) and the mean values were separated according to Tukey’s HSD test with *p* < 0.05. The following R packages were used for data visualization and statistical analyses: ggplot2, agricolae, ggpbur and Rcolorbrewer. The heatmap was generated using the “pheatmap” package. Hierarchical clustering was performed with Complete linkage and Euclidean distance was used as the similarity measure.

## Results

### Experiment 1: Phosphate solubilization and organic acids production by *Enterobacter* sp. strain 15S

**Fig. 2:**
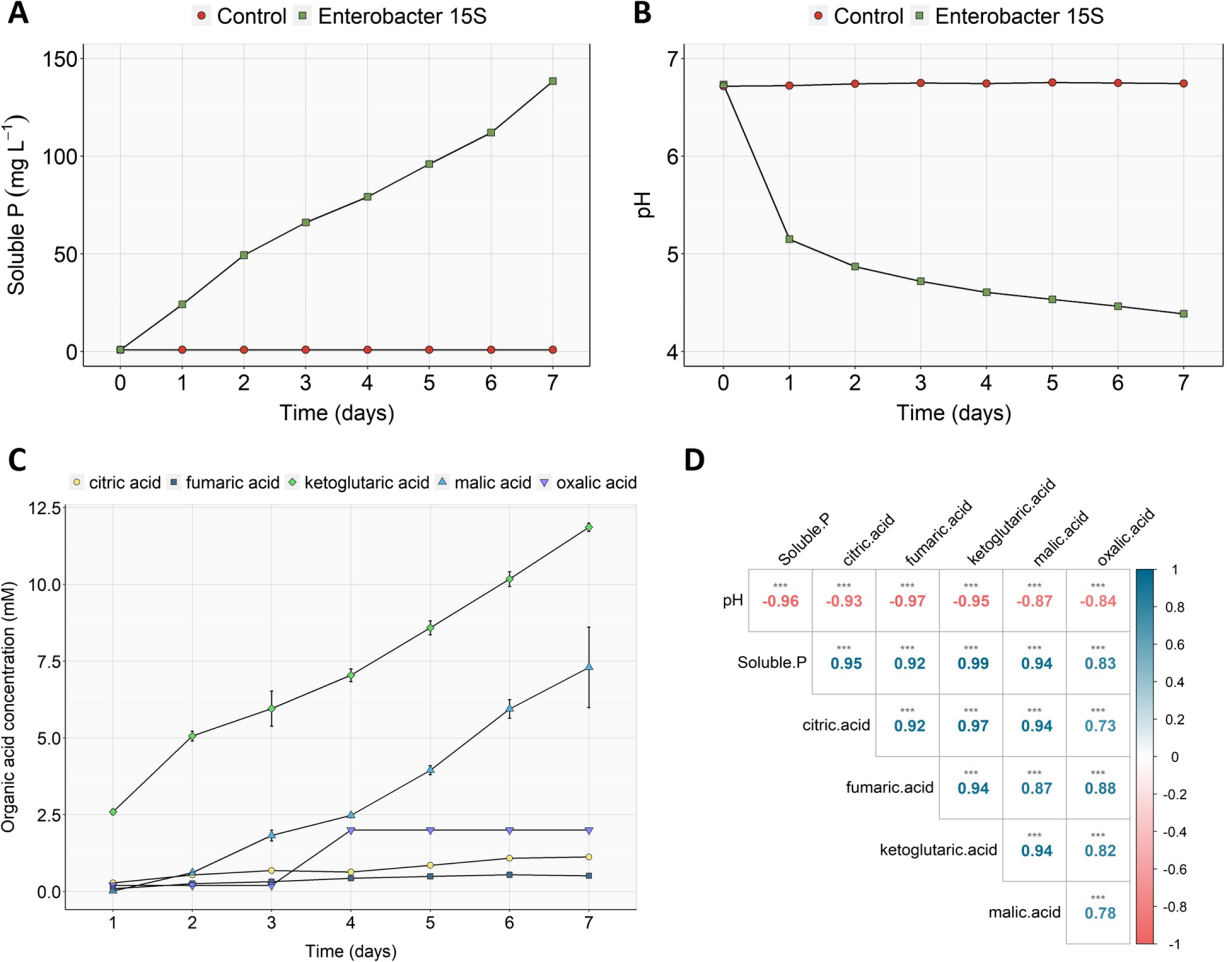
Mechanisms of P solubilization by *Enterobacter* 15S grown during 7 days in NBRIP liquid medium supplemented with Ca_3_(PO_4_)_2_ at 0.1% P (w/v). **A** Phosphate solubilization, **B** pH changes, **C** organic acid production, and **D** Pearson correlation matrix between solubilized P, pH values and organic acids. Color gradients and numbers denote Pearson’s correlation coefficients. Asterisks denote significance (*** *P* < 0.001)

*Enterobacter* 15S showed great ability in solubilizing Ca_3_(PO_4_)_2_. The available P (soluble P) estimated in the inoculated medium significantly increased during the incubation period, starting from 24 mg L^−1^ after 1 day and reaching the highest value of 138 mg L^−1^ after 7 days (Fig. 2A). In the first 2 days of culture, the bacterial strain showed its highest efficiency in solubilizing phosphate at a rate of 24 mg L^−1^ per day. Afterwards, the solubilization rate was kept at about 19–20 mg L^−1^ until the last day of culture. In the uninoculated control, solubilized phosphate was not detected in the medium (Fig. 2A).

The pH of inoculated medium significantly dropped to 5.1 on the first day (Fig. 2B) and gradually continued decreasing at an approximate rate of 0.15 pH units per day, reaching the value of 4.4 at the end of the experiment. On the other hand, the pH of uninoculated medium did not present any significant change along the incubation period (Fig. 2B).

The supernatants of the bacterial cultures were analyzed by HPLC in order to determine the qualitative and quantitative profile of OAs produced by the bacterial strain. During the solubilization of Ca_3_(PO_4_)_2_ by *Enterobacter* 15S, five OAs were identified and their concentrations showed a differential variation during the incubation time (Fig. 2C). For instance, the concentrations of α-ketoglutaric, malic, and citric acids linearly increased with the incubation period, whereas oxalic and fumaric acids production increased till day 4, and thereafter a plateau was reached. After 7 days of incubation in NBRIP medium, *Enterobacter* 15S produced predominantly α-ketoglutaric (12 mM) and malic (7 mM) acids, with concentrations significantly higher than those found for oxalic (2 mM), citric (1 mM) and fumaric (0.5 mM) acids.

The correlation between solubilized P, pH values and OAs was evaluated to explore the mechanism of P solubilization (Fig. 2D). Solubilized P and pH values showed a negative correlation (*r* = -0.96, *P* < 0.001) indicating that acidification is a key mechanism of *Enterobacter* 15S to dissolve phosphorus. Accordingly, a negative correlation between the pH value and the OAs content was also observed (*P* < 0.001). On the other hand, OAs concentrations and the soluble P fraction displayed a positive correlation (Fig. 2D), indicating that each OA produced by the bacterial strain play a specific role in solubilizing Ca_3_(PO_4_)_2_. In addition, acid and alkaline phosphatases activities were evaluated, but *Enterobacter* 15S did not show abilities to produce these enzymes (data not shown). These observations ruled out possible involvement of enzymatic P-solubilizing mechanism for *Enterobacter* 15S.

### Experiment 2: Effect of organic acids application on cucumber growth promotion and tricalcium phosphate solubilization

#### Plant growth and morpho-physiological traits

**Fig. 3:**
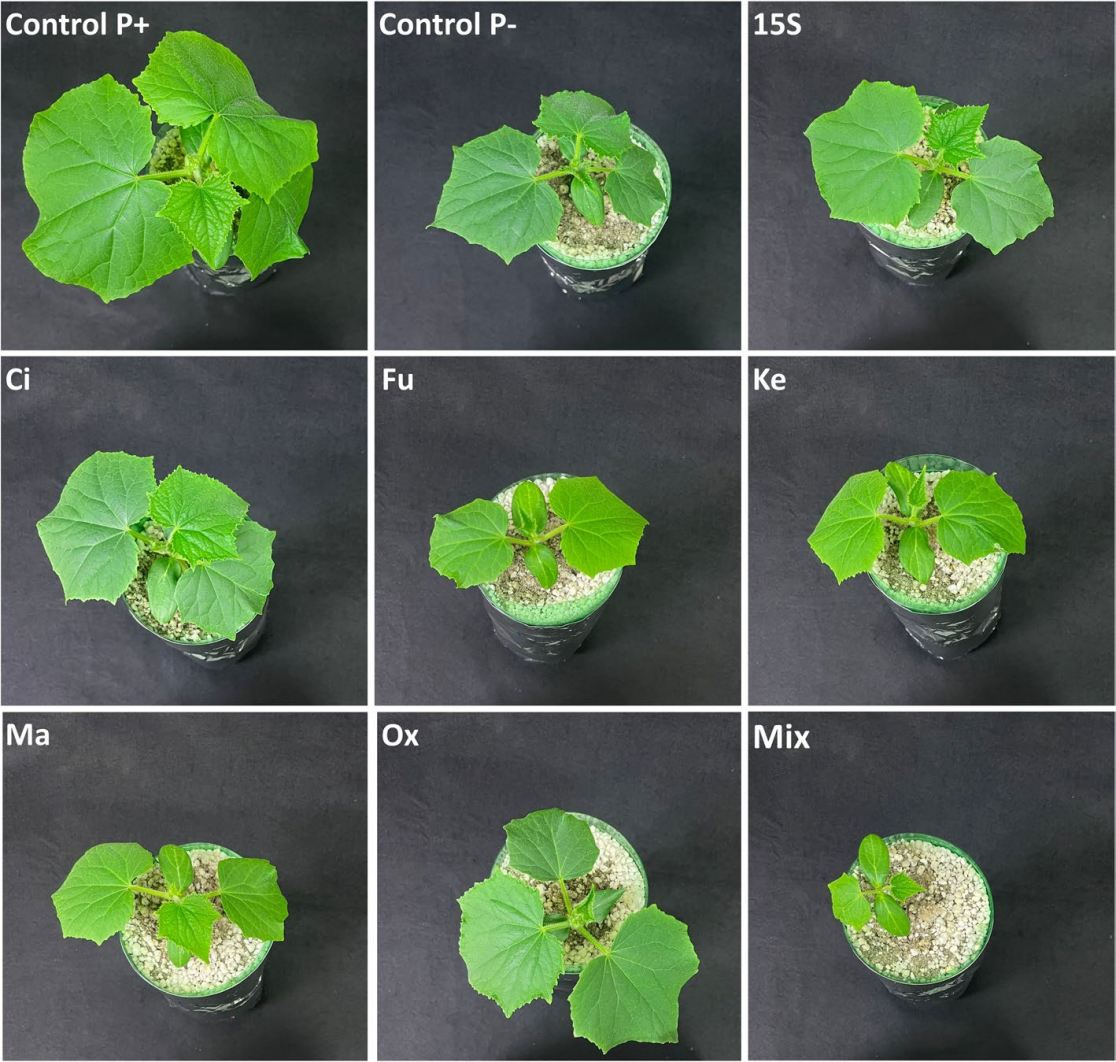
Representative pictures showing the growth of cucumber plants in the Leonard jars system upon the exogenous application of OAs produced by *Enterobacter* 15S. Pictures were taken at 21 days after transplanting

After 21 days of growth in the Leonard jars, cucumber plants were clearly affected by the different treatments (Fig. 3). Plants adequately fed with P (Control P +) presented the best shoot development with the highest leaf area and shoot biomass accumulation when compared to the other treatments (Table 2). Nonetheless, the inoculation with *Enterobacter* as well as the exogenous supplementation with OAs induced differential effects on the morpho-physiological traits of P-starved cucumber plants. In particular, treatments with citric and oxalic acids induced a higher shoot biomass accumulation (by 34%) and leaf area development (by about 20%) as compared to P-deficient Control treatment. Moreover, when inoculated with *Enterobacter* (15S treatment), plants showed a higher root biomass (20%), yet not significantly different compared to the Control P + , citric and oxalic acid treatments. Interestingly, the root/shoot (R/S) ratio was significantly higher in P-starved Control (P-) plants as compared to Control P + , whilst the 15S treatment induced the highest value. Furthermore, the highest SPAD index values were recorded in P-starved Control samples, whereas all the other plants presented lower SPAD values, regardless of the treatment. These results suggested that both bacterial inoculation and supplementation with OAs can be involved in the recovery from P-deficient plant symptoms. On the other hand, plants supplemented with the mix of OAs presented the worst development and, consequently, the lower values in all the morphological traits assessed (Table 2).

**Table 2:** Mean values for the morpho-physiological parameters in cucumber grown in the Leonard jars system under different treatments with OAs produced by *Enterobacter* 15S

Treatment	Parameters^a^				
	**RDW**	**SDW**	**R/S ratio**	**Leaf area**	**SPAD**
Control P +	0.17 ± 0.02 ab	0.44 ± 0.03 a	0.39 ± 0.03 c	184.21 ± 6.79 a	38.27 ± 0.51 de
Control P-	0.15 ± 0.01 bc	0.22 ± 0.01 c	0.70 ± 0.07 ab	81.13 ± 3.81 c	50.97 ± 0.15 a
15S	0.20 ± 0.01 a	0.23 ± 0.01 c	0.86 ± 0.03 a	77.86 ± 1.15 c	42.30 ± 0.36 c
Ci	0.18 ± 0.01 ab	0.30 ± 0.01 b	0.60 ± 0.04 b	95.09 ± 1.53 b	43.60 ± 0.36 b
Fu	0.13 ± 0.01 c	0.17 ± 0.01 d	0.76 ± 0.09 ab	39.87 ± 1.90 e	37.30 ± 0.30 fg
Ke	0.16 ± 0.02 bc	0.21 ± 0.01 c	0.75 ± 0.11 ab	65.91 ± 3.02 d	36.63 ± 0.152 g
Ma	0.16 ± 0.01 bc	0.21 ± 0.01 c	0.78 ± 0.08 ab	58.99 ± 3.64 d	39.10 ± 0.40 d
Ox	0.18 ± 0.00 ab	0.30 ± 0.01 b	0.60 ± 0.02 b	99.75 ± 2.28 b	42.97 ± 0.15 bc
Mix	0.03 ± 0.00 d	0.08 ± 0.01 e	0.34 ± 0.02 c	7.56 ± 0.76 f	37.97 ± 0.21 ef

Measurements have been carried out on three independent biological replicates (*n* = 3) and reported as means ± standard errors. Differences between means were determined using Tukey’s HSD test. Significant differences (*P* < 0.05) according to Tukey’s test are indicated by different lowercase letters within columns

^a^RDW (g plant^−1^): root dry weight; SDW (g plant^−1^): shoot dry weight; R/S: root-to-shoot ratio; Leaf area (cm^2^); SPAD units: soil–plant analysis development

#### Ionomic analysis

The whole ionome profile of roots and leaves of cucumber was analyzed through ICP-MS, including a set of five macronutrients (*i.e.*, phosphorus – P, potassium – K, calcium – Ca, magnesium – Mg and sodium—Na) and five micronutrients (*i.e.*, iron – Fe, zinc – Zn, copper – Cu, manganese – Mn and molybdenum—Mo). As expected, P-starved plants presented a decreased P concentration in roots and leaves compared to Control P + plants (Table S3). However, the treatment with *Enterobacter* 15S significantly increased P accumulation in the roots when compared to the other P-free treatments. Nonetheless, considering the plants supplemented with OAs, the exogenous application of oxalic acid was also effective in inducing a better P accumulation in both roots and leaves.

Phosphorus starvation induced an imbalanced distribution of the other macro- and micronutrients in cucumber plants, although a differential accumulation of these elements was observed depending on the treatment imposed (Table S3). For instance, in both roots and leaves, the concentration levels of K decreased, those of Ca and Mg increased, whilst Na content was unaffected. Howbeit, as a general trend, the supplementation with the mix of OAs induced the most contrasting accumulation of macronutrients compared to Control P + plants, increasing the concentration of Na, Mg, and Ca by about 2 up to 4 times. Regarding the accumulation of micronutrients, P-starvation induced an increased accumulation of Fe and Mn in roots as well as of Mo in leaves; on the other hand, Zn and Cu concentrations were unaffected in both plant tissues as compared to Control P + plants. However, no significant differences were observed among the different treatments (*i.e.*, OAs and 15S), except for plants treated with the mix of OAs, which induced an overaccumulation of Cu, Zn, and Mn up to 250% in roots and 70% in leaves (Table S3).

**Fig. 4:**
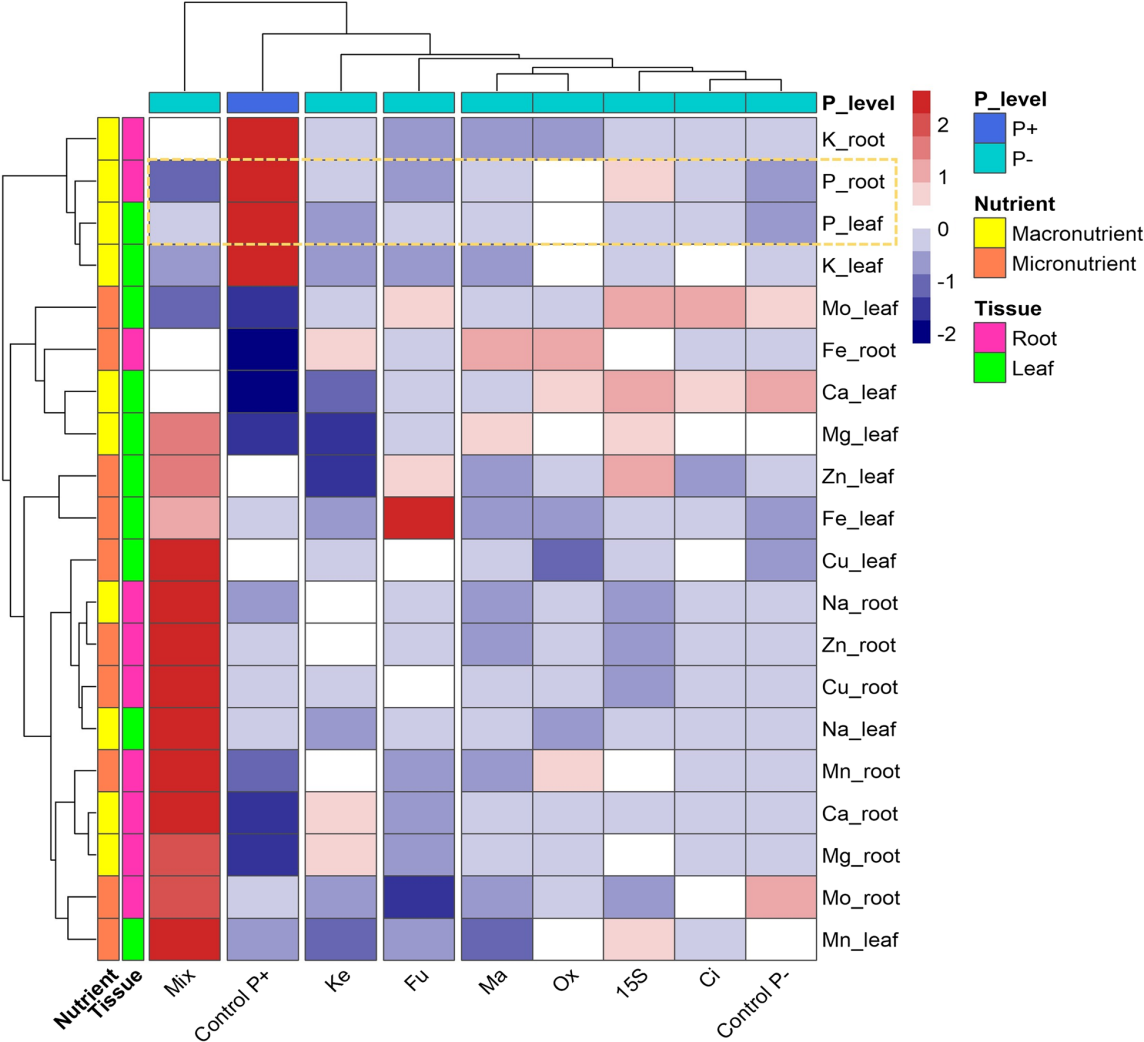
Cluster heatmap analysis displaying the macro- and micronutrients allocation in cucumber plants grown in the Leonard jars system and subjected to different treatments (i.e., OAs supplementation or *Enterobacter* 15S inoculation). Dark red color indicates higher relative values, while a dark-blue color reflects lower relative values. White color denotes values close or equal to zero, i.e., close or equal to the mean for each parameter

Furthermore, to better understand the influence of the different treatments on uptake and allocation of mineral nutrients, a cluster analysis was performed (Fig. 4). The treatments were grouped in five clusters, one encompassing five treatments (*i.e.*, malic, oxalic, 15S, citric, and Control P-) and four individual clusters for the other treatments (*i.e.*, Mix, Control P + , ketoglutaric, and fumaric). Clearly, the higher concentration of nutrients in roots was the main factor determining the separation of plants treated with the mix of OAs, suggesting a possible toxic effect driven by the high final concentration of OAs. The second cluster, encompassing Control P + plants, separated from the other treatments mainly because of the higher concentration of P and K in plant tissues (Fig. 4). The clustering of plants treated with ketoglutaric acid was driven by the lower concentration of Ca, Mg, Zn, and Fe in the leaves, whilst that of plants supplemented with fumaric acid was determined by increased Zn and Fe concentration in leaves. In the fifth cluster, the treatments determined a more balanced allocation of minerals. However, considering the effect on P nutrition, the inoculation with *Enterobacter* 15S induced a higher allocation of P in roots, whilst P-starved Control plants presented the lowest concentrations of P in both plant tissues. These observations suggest that, at least in the present experimental conditions, both *Enterobacter* 15S and its OAs improved P nutrition of cucumber plants, albeit to different extents (Fig. 4).

#### P-efficiency indexes

To determine the amount of P in the substrate that can be available for plants, an experiment without plants has been carried out and the soluble P was estimated after 21 days of treatment. Considering that the initial amount of P added was equal to 1000 mg kg^−1^ (either as Ca_3_(PO_4_)_2_ or as KH_2_PO_4_ depending on the treatment), a differential P solubilization was observed in *Enterobacter* 15S inoculated and OAs treated substrates (Table 3). For instance, the supplementation with the mix of OAs allowed the higher P release (29%), followed by the treatments with oxalic, malic ketoglutaric, citric acids and 15S, solubilizing 26%, 23%, 22%, 19%, 18% and 15% of P, respectively. In Control P- samples, a negligible amount of P (0.4%) was released, whilst almost all the P added into the P-full jars (Control P +) was present in the accessible form (about 93%).

**Table 3:** P-efficiency indexes as a function of the inoculation with *Enterobacter* 15S or the exogenous application of OAs

Treatment	P indexes^a^				
	**Available P**	**PAR**	**PAS**	**PUE**	**AREP**
Control P +	930.76 ± 15.16 a	3.21 ± 0.14 a	5.23 ± 0.03 a	21.12 ± 1.53 c	0.53 ± 0.01 a
Control P-	4.10 ± 0.03 g	0.79 ± 0.05 e	0.32 ± 0.04 fg	NA	NA
15S	149.84 ± 3.66 f	2.24 ± 0.07 b	0.57 ± 0.06 d	6.14 ± 1.10 d	0.19 ± 0.00 c
Ci	183.53 ± 1.20 e	1.52 ± 0.02 c	0.73 ± 0.01 c	39.01 ± 0.84 a	0.23 ± 0.00 c
Fu	190.15 ± 6.45 e	0.49 ± 0.03 f	0.37 ± 0.01 ef	-28.67 ± 4.25 f	0.04 ± 0.01 d
Ke	221.68 ± 7.19 d	1.27 ± 0.05 d	0.43 ± 0.00 def	-1.45 ± 0.91 e	0.05 ± 0.00 d
Ma	227.42 ± 3.98 d	1.21 ± 0.13 d	0.49 ± 0.02 de	-4.72 ± 0.87 e	0.08 ± 0.00 d
Ox	255.22 ± 9.38 c	1.72 ± 0.05 c	1.09 ± 0.13 b	28.50 ± 0.54 b	0.30 ± 0.05 b
Mix	294.19 ± 4.73 b	0.11 ± 0.01 g	0.23 ± 0.01 g	-47.20 ± 1.85 g	-0.03 ± 0.00 e

Means followed by the same lowercase letter in the column do not differ from each other by Tukey’s test (*P* < 0.05)

^a^Available P (mg kg^−1^): available P in the substrate (without plant) after 21 days of treatment; *PAR (mg)* Phosphorus accumulated in roots, *PAS (mg)* Phosphorus accumulated in shoots, *PUE* Phosphorus utilization efficiency, *AREP* Apparent recovery efficiency of phosphorus. NA: value not applicable for Control P- treatment

To further understand the relationship between the amount of P released by the treatments and the P use efficiency in cucumber plants, different indexes were calculated. Hence, the highest amount of P accumulated in roots (PAR) and shoots (PAS) was observed in P sufficient plants (Table 3). However, under P-free conditions, the supplementation with *Enterobacter* 15S, oxalic and citric acid induced a significantly higher accumulation of P in roots (by 50–65%) and shoots (by 45–70%) when compared to Control P- (Table 3). Conversely, plants treated with the mix of OAs presented the lowest accumulation of P, reduced by 7.4-fold in roots and 1.4-fold in shoots, with respect to Control P- plants.

The P utilization efficiency (PUE) was estimated by the relationship between shoot biomass with and without P and the amount of available P. Interestingly, the exogenous application of oxalic and citric acids induced a higher PUE (84% and 35%, respectively) when compared to Control P + samples, suggesting that plants treated with these two OAs were more efficient in using the uptaken P. On the other hand, since shoot biomass of plants treated with fumaric, ketoglutaric, malic, and mix of OAs was lower than the biomass of untreated plants (Control P-) a negative PUE was observed. Nonetheless, although a lower aboveground biomass per unit of available P (a lower PUE) was observed in plants inoculated with *Enterobacter* 15S, the use of this bacterial inoculant was still positive in improving PUE.

Regarding the apparent recovery efficiency of P (AREP – *i.e.*, the amount of P accumulated in shoots per unit of available P) different values were observed among the treatments (Table 3). The highest AREP was shown by Control P + plants, whilst a negative value was observed in plants treated with the mix of OAs. Nonetheless, among P starved plants, the supplementation with oxalic acid induced the highest AREP value, followed by those observed in plants treated with citric acid and *Enterobacter* 15S. Concerning fumaric, ketoglutaric and malic acids-treated plants, significantly lower AREP values were recorded. These results suggest that the uptake and mobilization of P were driven by the treatment applied rather than the availability of P in the substrate. Overall, under P-deficiency conditions, the P-efficiency indexes were higher in plants supplemented with oxalic acid, citric acid, or *Enterobacter* 15S, disclosing the best potentialities for these treatments in ameliorating P uptake and use.

#### Phosphate transporter gene expression

**Fig. 5:**
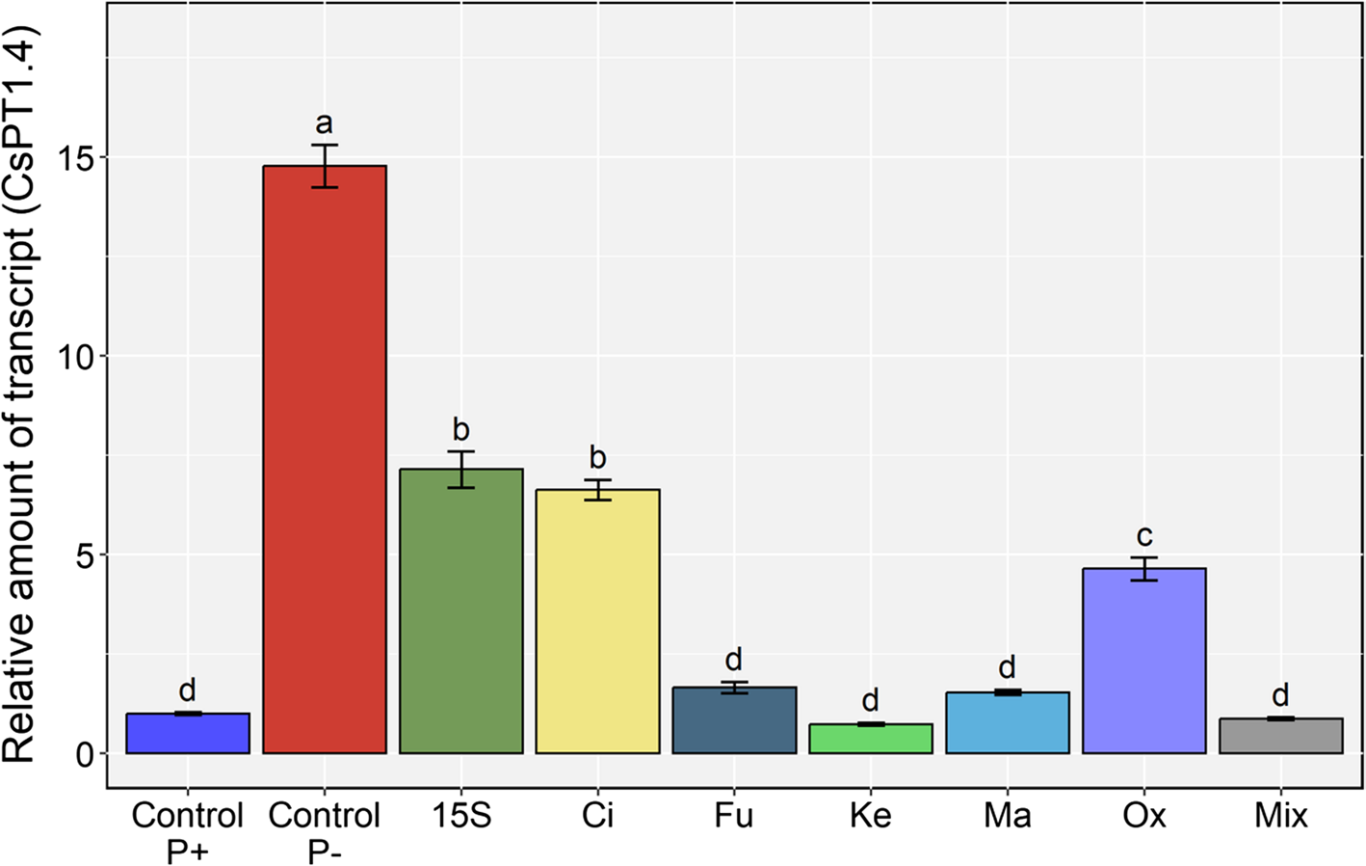
Gene expression analysis of *CsPT1.4* in the roots of cucumber grown in the Leonard jars system and subjected to different treatments (*i.e.,* OAs supplementation or *Enterobacter* 15S inoculation). The expression levels of *CsPT1.4* was normalized to the expression levels of the *elongation factor isoform 1-alpha* (*EF-1α*). The relative expression ratios were calculated using the P-full control as a calibrator sample. Values are means ± SE; *n* = 3. Equal letters correspond to average values that do not differ according to Tukey’s HSD test (*p* < 0.05)

The transcriptional modulation of *CsPT1.4*, a P transporter gene belonging to the *PHT1* family and highly induced in roots of P-deficient cucumber plants [9], was also investigated. The *CsPT1.4* gene was highly up-regulated in P-starved plants (Control P-), yet the bacterial inoculant or the exogenous application of OAs differentially affected the transcript abundance (Fig. 5). In plants inoculated with *Enterobacter* 15S or supplemented with citric acid, *CsPT1.4* was reduced by 50% as compared to Control P-, whereas it was reduced by about 70% oxalic acid-treated cucumber plants. On the other hand, the supplementation with fumaric, ketoglutaric, malic, and the mix of OAs did not induce any transcriptional modulation as compared with Control P + plants. Overall, our results suggest that organic acids produced by *Enterobacter* 15S and the bacterium itself had the potential to induce a greater P-uptake capacity in cucumber plants.

## Discussion

Several strains of *Enterobacter* have been described for their ability to solubilize significant amounts of tricalcium phosphate (TCP) in the NBRIP medium [40, 41]. In particular, *Enterobacter* sp. strain 15S showed a great potential in solubilizing TCP (138 mg L^−1^) after 7 days of culture. Interestingly, the negative correlation observed between pH values and the amount of soluble P in the culture medium suggested that acidification can play an important role in the *Enterobacter*-mediated P solubilization. In fact, it has been reported that the main mechanism used by PSB to dissolve insoluble P is the lowering of soil pH by either the production of organic acids (OAs) or the direct release of H^+^ [20]. In the present study, produced by *Enterobacter* 15S was shown to predominantly produce five OAs, namely citric, fumaric, ketoglutaric, malic, and oxalic acids. Indeed, the release of OAs for the TCP solubilization has already been suggested for different *Enterobacter* strains. For instance, P-solubilizing activity of *E. hormaechei* PSB6 was predominantly associated with the production of gluconic, glutamic, ketoglutaric, pyruvic, oxaloacetic, malic, succinic, acetic, and fumaric acids [42], whilst a mixture of gluconic, acetic, citric, malic, oxalic and succinic acids was identified in *E. cloacae* NG-33 during TCP solubilization in NBRIP medium [43]. Nonetheless, the efficacy of PSB in P solubilization depends on the amount and type of organic acids released, as well as on the bacterial strain [44]. In the present study, the P solubilization ability of *Enterobacter* 15S positively correlated with the levels of ketoglutaric and malic acids released, suggesting the crucial role of these two carboxylates in the process evaluated in vitro conditions.

When the process was tested in situ in a P-free soil–plant system, treatments-dependent effects were observed on plants morpho-physiological parameters. Indeed, the exogenous application of oxalic and citric acids, as well as the inoculation with *Enterobacter* 15S induced the most remarkable effect on plant development as compared to Control P-. Inoculation with PSB is considered a promising tool to promote plant growth and yield. These effects are produced by an increased P uptake at root level, that indeed results from OAs secretion by microbes and the P solubilizing property of such carboxylates [45]. Accordingly, citric acid (2.5 mM) supplementation in soil has already been shown to enhance P uptake and the yield of tomato plants [31]. On the contrary, the supply of OAs mixture severely inhibited the cucumber plant growth, suggesting a possible toxic effect maybe ascribable to the final OAs concentration. In fact, it has already been reported that OAs concentration higher than 6 mM can induce phytotoxic effect, as for instance stunted plant growth [46, 47].

Regarding root development, cucumber plants allocated more biomass belowground, thus resulting in a higher R/S ratio under P starvation. It is interesting to note that *Enterobacter* 15S further exacerbated this effect. In a more general context, the effect of P deficiency on root development and architecture is complex and involves a crosstalk between different hormone-signaling pathways, generally leading to an enhanced formation of lateral roots and root hairs [48]. Nonetheless, a larger root system was also expected in the treatment with *Enterobacter* 15S, which has previously been shown to release auxins in the rhizosphere [9, 13]. Beside root system, P deficiency also affects shoot, by limiting its development and inducing a darkening of leaves, as a consequence of anthocyanin accumulation [49]. Interestingly, in our experimental system, either the inoculation with *Enterobacter* 15S or the supplementation with selected OAs induced a clear decrease in the SPAD index. This result, together with the increased P concentrations measured in plant tissues, suggests a partial recovery from P deficiency in treated plants.

Phosphorus starvation can alter the concentration levels of either essential or non-essential nutrients, resulting in physiological, morphological and metabolic disorders in plants [9]. In the present study, P shortage caused a decrease of P and K concentrations in roots and shoots, whilst an increase in those of Ca and Mg was observed. With respect to Na, Zn, and Cu, their levels remained unaffected by the treatments. Additionally, Fe and Mn concentrations increased only in the roots of P-deficient plants. It is well known that the dynamics in the functional ionome of plants subjected to individual mineral deficiency indicate the existence of complex relationships between macro- and micronutrients [50, 51]. For instance, several pieces of evidence indicate that P deficiency induces an increased Fe concentrations in plant tissues [52, 53], whilst other nutrients show more variable responses to P deficiency [54]. On the other hand, the use of PSB together with sparingly soluble P forms has been described as an integrated plant nutrition system that may lead to a successful bacterium-plant association by improving P availability and P mineral nutrition [55]. Accordingly, maize and wheat plants fertilized with rock phosphate and inoculated with strains of *Pantoea* or *Pseudomonas* showed a higher allocation of P in roots and shoots [56], as well as in cucumber inoculated with *Enterobacter* strain 15S in combination with TCP [9].

Furthermore, the soil supplementation with individual OAs could significantly affect mineral acquisition. In fact, the heatmap analysis showed that the exogenous application of *Enterobacter*-secreted OAs induced a differential pattern in the accumulation of mineral nutrients in cucumber plants. The most remarkable effect was observed with the mix of OAs, which induced an overallocation of nearly all macro- and micronutrients in the roots, indicating that the elevated concentration of OAs in this treatment could have promoted a severe disturbance in the homeostasis of plant nutrients. Indeed, the hyperaccumulation of micronutrients has caused the overcoming of a concentration threshold, above which toxic effects could be induced on plants, as also demonstrated by the phenotypic observations [57]. In this context, such high availability of metals in the rhizosphere has been demonstrated to have detrimental effects on the biochemical and molecular mechanisms underpinning macronutrients (*e.g*., P, N) acquisition [58–61]. On the other hand, a contrasting effect regarding the accumulation of Zn and Fe in cucumber leaves was induced by ketoglutaric and fumaric acids. The ability of OAs to dissolve insoluble minerals in the soil is mainly attributed to acidification and chelation processes, which facilitate metals solubility and, ultimately, boost mineral acquisition by plants [62]. However, stimulating effects on the metals acquisition have been usually reported at low concentrations of OAs, whereas, at high concentrations, OAs may cause phytotoxic effects [63]. For instance, the addition of citric acid with the irrigation water increased the concentration of Ni, Zn, Co, Cr, Mn, and Fe in leaves of *Noccaea caerulescens* [64], whereas the same OA decreased the concentration of Zn and Mn in tomato leaves [31]. Overall, these observations suggest that the modulations of plant ionomic signature in response to OAs application are dependent on concentration, strength, and type of OA type, as well as on plant species.

The hypothesis that *Enterobacter* 15S solubilizes TCP mainly via OAs secretion was further confirmed in the plant-free experiment under in situ conditions. In this experiment, it has been showed that the individual application of the five OAs produced by *Enterobacter* 15S, the mixture of OAs, and the inoculation with the bacterium itself were effective in the solubilization of TCP. Nonetheless, the OAs produced at higher concentrations under in vitro conditions were not correlated to the higher P release (available P) under in situ conditions, possibly suggesting that strength and type, rather than concentration, could play a key role in determining OAs ability to mobilize P from soil pools. One of the most important mechanisms by which microbial-secreted OAs solubilize P is the chelation of cations bound to phosphate through their hydroxyl and carboxyl groups. Thus, the number of carboxylic groups and the acidity constants (pKa values) have been considered determinants in the efficacy of P solubilization [65]. For instance, oxalic acid (pKa1: 1.27, pKa2: 4.28) has been reported to be more effective than citric acid (pKa1: 3.13, pKa2: 4.76, pKa3: 6.40) in releasing P from calcareous soils because oxalate can form a stronger and more stable complexes with calcium [66]. Interestingly, by determining the pH values of the growth substrate (Fig. S1), we could not highlight any significant alterations according with the treatment imposed. These observations further support the hypothesis that P mobilization preferentially occurs via chelation, at least in the in situ experimental setup.

Regarding the amount of P accumulated in roots (PAR) and shoots (PAS), the higher were observed in plants either inoculated with *Enterobacter* 15S or supplemented with oxalic and citric acids, whilst the lowest P accumulation was detected in plants treated with the mixture of OAs. The exogenous application of oxalic acid significantly enhanced P acquisition in maize [32] and rice [30], while citric acid and malic acids had no significant impact. These results further corroborate the role of OAs in P mobilization at rhizosphere level, albeit being extremely dependent on the soil type, the inorganic phosphate source, the plant species, and the specific type of OA applied. Additionally, plants treated with oxalic and citric acids were more efficient in utilizing the available P released from TCP, contributing to a higher biomass production, which was translated into a higher PUE, as also previously observed [67]. Moreover, despite showing lower levels as compared to oxalic and citric acid treatments, plants inoculated with *Enterobacter* 15S displayed positive PUE values. A similar trend was also recorded for the apparent recovery efficiency (AREP), where oxalic acid induced the highest value, followed by citric acid and the bacterium. Indeed, the achievement of higher PUE in agricultural crops by applying eco-friendly approaches, as PSB, is becoming mandatory to guarantee, on one hand, the productivity and, on the other hand, the sustainability of the cropping system [45].

Under P deficiency, members of the high affinity transport system (*PHT1*), expressed at root levels, are involved in P translocation from the extracellular environment into the cytoplasm [68]. Although six *PHT1* genes have been identified in cucumber, namely *CsPT1.3*, *CsPT1.4*, *CsPT1.7*, *CsPT1.9*, *CsPT1.11*, and *Cucsa383630.1* [58], we focused on the modulation of *CsPT1.4* expression, since it was previously shown to be strongly expressed in roots of cucumber under P-deficiency [9]. In Control P- plants, *CsPT1.4* transcript levels were highly induced in cucumber roots, whilst a drop in the relative expression was observed following the treatments with *Enterobacter* 15SS, citric and oxalic acids. Interestingly, as already mentioned, these same treatments promoted the highest P accumulation in roots under P deficiency, most likely through an efficient solubilization of P from TCP. Such increase in P bioavailability might be thus responsible for the down-regulation of *CsPT1.4* transcription in cucumber roots. Similar findings were described by Liu et al. [69] concerning the modulation of *TaPT4* in wheat roots inoculated with *Pseudomonas* strain P34-L. These results highlight the great potential of PSB to improve plant growth under impaired nutritional conditions. Moreover, the use of fumaric, ketoglutaric, and malic acids, as the mixture of OAs did not significantly influence the expression of *CsPT1.4* as compare to Control P + plants, possibly indicating an adequate P acquisition in these conditions. However, data of the morphologic and ionomic analysis do not fully support this hypothesis. Yet, previous studies demonstrated that that the imbalanced homeostasis of specific micronutrients interferes with the transcription levels of *PHT1* genes in cucumber roots [58].

## Conclusions

The use of PSB as biofertilizers has become a promising strategy to enhance crop production by improving plant P acquisition and, thereby, limiting the use of chemical fertilizers. The crucial role of the OAs released by PSB in the P-mobilization has been widely reported in experiments carried out in vitro. However, to date, there are no reports clarifying the role of the individual OAs produced by a particular bacterial strain in this process. In the present work, using a soil–plant system, it has been shown that *Enterobacter* 15S strain predominantly releases ketoglutaric and malic acids when cultivated under in vitro conditions. However, the in situ experiment showed that the treatments responsible for a better plant P nutrition were oxalic acid, citric acid and bacterial inoculation, that, overall, induced a positive PUE in cucumber plants. Noteworthy, *Enterobacter* 15S was able to induce the highest PAR in P starved plants. Most importantly, our experiment demonstrates that findings obtained in controlled conditions (*i.e.*, in vitro) might not necessarily reflect the events taking place at rhizosphere level. On the other hand, these data open a new perspective showing that the supplementation with specific OAs might represent a promising tool to ameliorate P nutrition in plants, exploiting P sources naturally occurring in soils. However, the implementation of such practices might not fulfill the requisite of both environmental and economical sustainability, thus requiring further research efforts. On the contrary, *Enterobacter* 15S can be accounted as the best performing treatment for the enhancement of P nutrition in cucumber plants, respecting the sustainability paradigm. Yet, in order to better exploit the potentialities of *Enterobacter* 15S as biofertilizers, further studies in larger scale (e.g., field conditions and/or different crops) need to be carried out.

## Supplementary Information

**Additional file 1: Table S1.** Texture and chemical composition of the sand without the supplementation with Ca_3_(PO_4_)_2_. (DW = dry weight).** Table S2.** Sequence of forward and reverse primers used in Real-time RT-PCR experiments. **Table S3.** Mean values for ionomic analysis of root and leaf tissues of cucumber grown in the Leonard jars system under different treatments with the organic acids produced by *Enterobacter* 15S. Differences between means were determined by Tukey’s HSD test. Different letters within the rows indicate statistically different values (*p* < 0.05).** Fig. S1. **pH values of the growth substrate after 21 days of treatment. Values are means ± SE; *n* = 3. Equal letters correspond to average values that do not differ according to Tukey’s HSD test (*p* < 0.05).

## Data Availability

The authors declare that the data supporting the findings of this study are available within the paper and its Supplementary Information files. Should any raw data files be needed in another format they are available from the corresponding author upon reasonable request.

## Abbreviations

OAs: Organic acids
PSB: Plant solubilizing bacteria
TCP: Tricalcium phosphate
P: Phosphorus
LB: Luria-Bertani medium
NS: Nutrient solution
SPAD: Soil-plant analysis development
RDW: Root dry weight
SDW: Shoot dry weight
qRT-PCR: Quantitative real-time PCR
PAR: Phosphorus accumulated in roots
PAS: Phosphorus accumulated in shoots
PUE: Phosphorus utilization efficiency
AREP: Apparent recovery efficiency of phosphorus
R/S: Root to shoot ratio
*PHT*: Phosphate transporter gene
