# Supplementary material for: An insight into the role of the organic acids produced by Enterobacter sp. strain 15S in solubilizing tricalcium phosphate: in situ study on cucumber
Source: BMC Microbiol. 2023 Jul 12;23:184. doi: 10.1186/s12866-023-02918-6 (PMC10337144; doi:10.1186/s12866-023-02918-6)
Supplement: Supplementary file 1 — Additional file 1: Table S1. Texture and chemical composition of the sand without the supplementation with Ca3(PO4)2. (DW = dry weight). Table S2. Sequence of forward and reverse primers used in Real-time RT-PCR experiments. Table S3. Mean values for ionomic analysis of root and leaf tissues of cucumber grown in the Leonard jars system under different treatments with the organic acids produced by Enterobacter 15S. Differences between means were determined by Tukey’s HSD test. Different letters within the rows indicate statistically different values (p < 0.05). Fig. S1. pH values of the growth substrate after 21 days of treatment. Values are means ± SE; n = 3. Equal letters correspond to average values that do not differ according to Tukey’s HSD test (p < 0.05). [file 12866_2023_2918_MOESM1_ESM.docx]

**Supplementary Information**

**Table S1**. Texture and chemical composition of the sand without the supplementation with Ca_3_(PO_4_)_2_. (DW = dry weight).

| Sand parameters |  | value | unit |
| --- | --- | --- | --- |
| Sand texture: |  |  |  |
| *Sand (2.000-0.050 mm)* |  | 94 | % |
| *Gravel (>2.000 mm)* |  | 4 | % |
| *Fines (<0.050 mm)* |  | 2 | % |
|  |  |  |  |
| pH |  | 8.06 |  |
| Organic matter |  | 0.09 | %DW |
| Organic carbon |  | 0.16 | %DW |
| Total N |  | 0.029 | %DW |
| Total P |  | 0.035 | %DW |
| Ca |  | 1651 | ppm |
| Cu |  | 1.4 | ppm |
| Zn |  | 0.9 | ppm |
| Fe |  | 2406 | ppm |

**Table S2**. Sequence of forward and reverse primers used in Real-time RT-PCR experiments.

| **Gene** | **Transcript accesion number** | **Forward primer 5'-3'** | **Reverse primer 5'-3'** |
| --- | --- | --- | --- |
| CsEF1α | XM_004138916.3 | ATTTGCTGTCCGTGATATGCG | CTTCTTCACAGCGGACTTGG |
| CsPT1.4 | XM_004134017.3 | TCACTTTGTTAGTTCCTGAATC | TCAAACAAGCACAGTTCTTGA |

**Table S3**. Mean values for ionomic analysis of root and leaf tissues of cucumber grown in the Leonard jars system under different treatments with the organic acids produced by *Enterobacter* 15S. Differences between means were determined by Tukey’s HSD test. Different letters within the rows indicate statistically different values (*p* < 0.05).

| **Elements** | **Tissue** | **Treatments** | | | | | | | | | | | | | | | | | | | | | | | | | | |
| --- | --- | --- | --- | --- | --- | --- | --- | --- | --- | --- | --- | --- | --- | --- | --- | --- | --- | --- | --- | --- | --- | --- | --- | --- | --- | --- | --- | --- |
|  |  | **Control P+** | | | **Control P-** | | | **15S** | | | **Ci** | | | **Fu** | | | **Ke** | | | **Ma** | | | **Ox** | | | **Mix** | | |
|  |  |  |  |  |  |  |  |  |  |  |  |  |  |  |  |  |  |  |  |  |  |  |  |  |  |  |  |  |
| P (mg g DW^-1^) | Root | 20.53 | ± | 0.10 a | 4.74 | ± | 0.33 g | 11.12 | ± | 0.28 b | 8.55 | ± | 0.42 d | 5.59 | ± | 0.26 f | 8.46 | ± | 0.19 d | 7.49 | ± | 0.26 e | 9.58 | ± | 0.11 c | 4.18 | ± | 0.13 g |
|  | Leaf | 13.11 | ± | 1.19 a | 1.42 | ± | 0.12 c | 2.45 | ± | 0.21 bc | 2.47 | ± | 0.04 bc | 2.12 | ± | 0.25 c | 1.99 | ± | 0.01 c | 2.52 | ± | 0.27 bc | 3.65 | ± | 0.34 b | 2.51 | ± | 0.32 bc |
| K (mg g DW^-1^) | Root | 12.25 | ± | 0.58 a | 4.21 | ± | 0.62 c | 4.12 | ± | 0.01 cd | 4.55 | ± | 0.55 bc | 3.04 | ± | 0.24 d | 4.35 | ± | 0.19 bc | 3.03 | ± | 0.09 d | 3.42 | ± | 0.59 cd | 5.48 | ± | 0.02 b |
|  | Leaf | 9.48 | ± | 0.63 a | 3.53 | ± | 0.27 c | 3.76 | ± | 0.12 bc | 3.85 | ± | 0.27 bc | 2.49 | ± | 0.30 d | 2.33 | ± | 0.18 d | 2.56 | ± | 0.05 d | 4.48 | ± | 0.25 b | 2.05 | ± | 0.39 d |
| Ca (mg g DW^-1^) | Root | 19.11 | ± | 2.11 d | 35.52 | ± | 2.30 bc | 34.53 | ± | 0.66 c | 32.52 | ± | 1.31 c | 29.71 | ± | 1.65 c | 43.34 | ± | 1.93 b | 30.74 | ± | 0.63 c | 34.16 | ± | 1.39 c | 60.84 | ± | 7.42 a |
|  | Leaf | 18.18 | ± | 2.80 e | 47.18 | ± | 1.81 ab | 49.79 | ± | 4.30 a | 44.84 | ± | 4.29 abc | 33.44 | ± | 2.92 cd | 26.09 | ± | 2.41 de | 35.59 | ± | 2.51 bcd | 43.28 | ± | 2.87 abc | 42.41 | ± | 10.28 abc |
| Mg (mg g DW^-1^) | Root | 6.75 | ± | 0.69 d | 13.10 | ± | 0.32 c | 13.39 | ± | 0.61 c | 12.59 | ± | 1.43 c | 11.21 | ± | 0.39 c | 15.96 | ± | 0.40 b | 12.62 | ± | 0.85 c | 11.87 | ± | 1.42 c | 21.76 | ± | 0.52 a |
|  | Leaf | 7.83 | ± | 0.31 d | 12.61 | ± | 1.18 ab | 12.66 | ± | 0.19 ab | 11.98 | ± | 0.90 ab | 11.35 | ± | 1.23 bc | 8.16 | ± | 0.44 cd | 12.74 | ± | 1.35 ab | 11.90 | ± | 0.81 b | 15.38 | ± | 2.59 a |
| Na (mg g DW^-1^) | Root | 13.74 | ± | 2.00 bc | 18.08 | ± | 0.42 bc | 11.60 | ± | 0.16 c | 14.96 | ± | 1.03 bc | 17.19 | ± | 0.37 bc | 20.42 | ± | 2.39 b | 13.38 | ± | 0.25 c | 15.06 | ± | 1.52 bc | 45.62 | ± | 6.02 a |
|  | Leaf | 8.22 | ± | 0.60 b | 8.06 | ± | 0.65 b | 10.15 | ± | 0.06 b | 7.54 | ± | 0.89 b | 8.31 | ± | 0.22 b | 6.31 | ± | 1.02 b | 6.88 | ± | 0.08 b | 6.30 | ± | 0.52 b | 34.47 | ± | 11.86 a |
| Fe (µg g DW^-1^) | Root | 2836.87 | ± | 340.50 d | 6771.20 | ± | 57.42 c | 7962.94 | ± | 715.26 bc | 6926.38 | ± | 523.66 c | 6662.32 | ± | 58.79 c | 9138.46 | ± | 1100.24 ab | 9619.70 | ± | 345.64 ab | 9899.12 | ± | 982.87 a | 8307.28 | ± | 771.29 abc |
|  | Leaf | 352.16 | ± | 11.58 bc | 302.29 | ± | 12.14 bc | 327.58 | ± | 22.13 bc | 318.74 | ± | 2.11 bc | 593.20 | ± | 42.29 a | 262.24 | ± | 15.02 c | 261.73 | ± | 4.78 c | 305.83 | ± | 54.71 bc | 453.96 | ± | 170.23 ab |
| Zn (µg g DW^-1^) | Root | 107.75 | ± | 10.59 bc | 99.59 | ± | 2.21 c | 95.42 | ± | 3.02 c | 100.55 | ± | 2.78 c | 102.43 | ± | 6.46 c | 134.85 | ± | 5.56 b | 92.50 | ± | 1.81 c | 104.85 | ± | 0.09 bc | 244.97 | ± | 29.19 a |
|  | Leaf | 83.41 | ± | 3.90 bcd | 79.50 | ± | 3.05 bcd | 94.27 | ± | 0.70 ab | 75.99 | ± | 0.99 cd | 92.57 | ± | 1.62 abc | 67.22 | ± | 1.85 d | 74.89 | ± | 3.28 d | 80.71 | ± | 0.17 bcd | 101.04 | ± | 16.54 a |
| Cu (µg g DW^-1^) | Root | 407.01 | ± | 21.14 b | 403.08 | ± | 7.81 b | 236.12 | ± | 13.16 b | 393.40 | ± | 28.42 b | 521.09 | ± | 33.16 b | 350.51 | ± | 31.91 b | 372.35 | ± | 16.36 b | 412.57 | ± | 1.87 b | 1405.88 | ± | 357.22 a |
|  | Leaf | 284.77 | ± | 20.87 b | 215.88 | ± | 13.47 b | 266.26 | ± | 14.33 b | 283.04 | ± | 9.14 b | 306.18 | ± | 34.71 ab | 249.64 | ± | 19.82 b | 250.75 | ± | 13.07 b | 200.74 | ± | 36.29 b | 486.08 | ± | 191.73 a |
| Mo (µg g DW^-1^) | Root | 1.61 | ± | 0.11 d | 2.34 | ± | 0.04 b | 1.49 | ± | 0.15 d | 2.00 | ± | 0.24 bc | 0.96 | ± | 0.11 e | 1.50 | ± | 0.12 d | 1.48 | ± | 0.01 d | 1.72 | ± | 0.12 cd | 3.05 | ± | 0.14 a |
|  | Leaf | 3.15 | ± | 0.30 d | 7.67 | ± | 0.08 ab | 8.34 | ± | 0.41 a | 8.82 | ± | 0.20 a | 8.07 | ± | 0.06 a | 6.00 | ± | 0.13 c | 6.46 | ± | 1.33 bc | 6.04 | ± | 0.38 c | 4.06 | ± | 0.51 d |
| Mn (µg g DW^-1^) | Root | 143.46 | ± | 3.68 d | 204.12 | ± | 1.04 bc | 224.12 | ± | 3.12 bc | 205.49 | ± | 15.36 bc | 177.88 | ± | 1.35 cd | 231.14 | ± | 32.32 bc | 186.72 | ± | 8.49 cd | 255.81 | ± | 26.75 b | 355.65 | ± | 37.41 a |
|  | Leaf | 70.00 | ± | 3.63 b | 81.72 | ± | 1.71 b | 89.32 | ± | 2.86 ab | 77.25 | ± | 1.74 b | 62.04 | ± | 2.02 b | 60.79 | ± | 1.36 b | 60.65 | ± | 1.32 b | 83.46 | ± | 6.21 b | 124.26 | ± | 37.43 a |


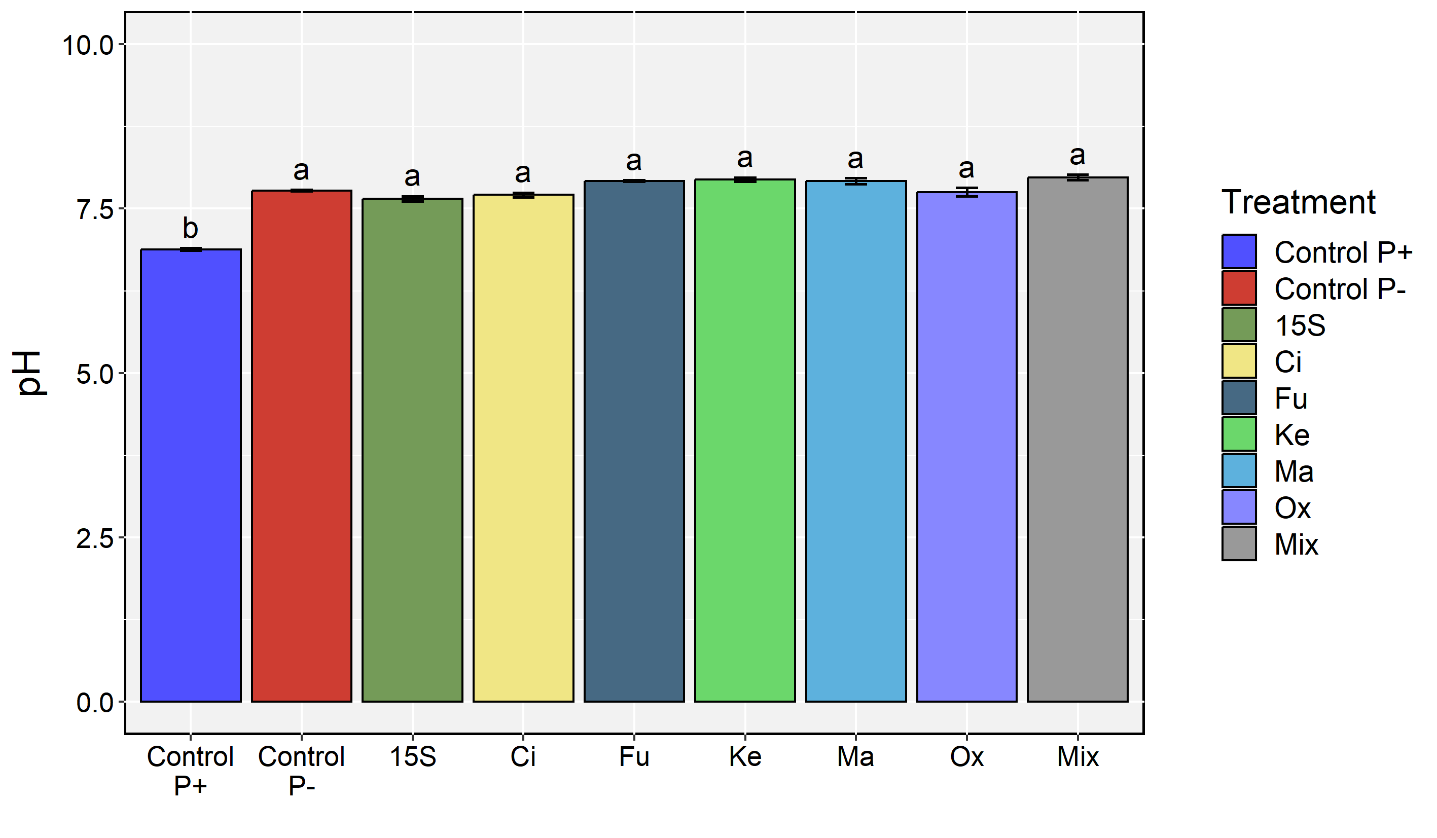


**Fig. S1** pH values of the growth substrate after 21 days of treatment. Values are means ± SE; n = 3. Equal letters correspond to average values that do not differ according to Tukey’s HSD test (p < 0.05).
